# Skin/muscle incision and retraction regulates the persistent postoperative pain in rats by the Epac1/PKC-βII pathway

**DOI:** 10.1186/s12871-022-01771-w

**Published:** 2022-07-18

**Authors:** Jiashu Qian, Xuezheng Lin, Zhili Zhou

**Affiliations:** Department of Anesthesiology, Taizhou Central Hospital (Affiliated Hospital of Taizhou University), No. 999 Donghai Avenue, Jiaojiang Economic Development Zone, Taizhou City, 318000 Zhejiang Province China

**Keywords:** Persistent postoperative pain, Skin/muscle incision and retraction, Epac1/PKC-βII

## Abstract

Persistent postoperative pain causes influence the life quality of many patients. The Epac/PKC pathway has been indicated to regulate mechanical hyperalgesia. The present study used skin/muscle incision and retraction (SMIR) to induce postoperative pain in rats and evaluated the Epac/PKC pathway in postoperative pain. Mechanical allodynia was assessed by paw withdrawal threshold before and after incision. The levels of Epac, PKC, proinflammatory cytokines, and blood-nerve barrier-related proteins were assessed using Western blotting. We found that SMIR induced the activation of the Epac/PKC pathway, mechanical allodynia, and upregulation of Glut1, VEGF, and PGP9.5 proteins in dorsal root ganglia. Under the influence of agonists of Epac/PKC, normal rats showed mechanical allodynia and increased Glut1, VEGF, and PGP9.5 proteins. After inhibition of Epac1 in rats with SMIR, mechanical allodynia was alleviated, and proinflammatory cytokines and Glut1, VEGF, and PGP9.5 proteins were decreased. Moreover, dorsal root ganglia neurons showed abnormal proliferation under the activation of the Epac/PKC pathway. Using Captopril to protect vascular endothelial cells after SMIR had a positive effect on postoperative pain. In conclusion, SMIR regulates the persistent postoperative pain in rats by the Epac/PKC pathway.

**Supplementary Information:**

The online version contains supplementary material available at 10.1186/s12871-022-01771-w.

## Introduction

Pain refers to a conscious unpleasant sensory and emotional experience associated with actual or potential tissue damage [1]. Pain sensation results from complicated mechanisms underlying all the nervous systems, from the periphery, throughout the spinal cord, to the cerebral structures [2]. Patients undergo postoperative pain due to the surgical procedure, preexisted diseases or a combination of the above-related causes [3]. Postoperative pain is deemed as the cause of multiple postoperative complications, and pain relief after surgery remains a major medical challenge [4]. A recent study suggested that 75% of patients who had received major surgery experienced postoperative pain, and 70% of them claimed to have unbearable pain despite taking analgesics [5]. For humanitarian reasons and to decrease postoperative morbidity and mortality, improvement in postoperative analgesia is essential [6]. Methods to control postoperative pain include pharmacologic treatment, anesthesia, cognitive treatment, and behavior methods [7].

Prostaglandin E2 is the best known lipid mediator that contributes to inflammatory pain, which leads to mechanical hyperalgesia by the cAMP/PKCε pathway after inflammation [8]. Under different conditions, cAMP stimulates Epacs to activate small Ras family of G proteins [9, 10], and activates many effector proteins, including PKCε and MAP kinases to facilitate various pathological states, including chronic pain [11–13]. Epac can mediate the activation of PKCε to cause mechanical hyperalgesia.

Maximum plasma levels of proinflammatory cytokines including TNFα, IL-1β, and IL-6 were immediately found after surgery [14]. Moreover, alterations of the blood-nerve barrier (BNB) functions are associated with nerve injury [15]. Besides, this homeostatic barrier disrupts the healing process after injury [16] and also regulates nerve sensitization [17]. A recent study revealed the implication of vascular endothelial growth factor (VEGF) in BNB opening [18].

In the present study, we used a rat model of persistent postoperative pain to evaluate the role of the Epac/PKC pathway in postoperative pain. Effects of this pathway in the expression of inflammation and BNB-related proteins and mechanical hyperalgesia were assessed, which can provide a new insight into the pathogenesis of postoperative pain.

## Materials and methods

### Animals

Adult male Sprague–Dawley rats weighing at 200–240 g were housed on sawdust bedding in plastic cages with available food and water *ad libitum*. Animals were kept under 50% humidity at 25 ± 2°C in a 12 h light and 12 h dark cycle. All the animal studies were performed following the guidelines of the Association for the Study of Pain and were approved by the Institutional Animal Care and Use Committee of Taizhou Central Hospital. Skin/muscle incision and retraction (SMIR) was operated in the thigh to establish a rat model of persistent postoperative pain, akin to a clinical procedure. Under anaesthesia, skin and superficial muscle of the medial thigh were incised and a small pair of retractors inserted. This tissue was retracted for 1h causing potential stretch of the saphenous nerve [19]. The SMIR model can evaluate pain behavior by hind paw stimulation and prolonging tissue retraction. Persistent mechanical hypersensitivity was evoked in more than 80% of SMIR-operated rats.

### Animal grouping

Rats were assigned into eleven groups: (i) the control group: rats received no treatment; (ii) the sham group: rats were sham-operated; (iii) the 0.25% DMSO group: rats were intraperitoneally injected with 0.25% DMSO; (iv) the PMA group: rats were intraperitoneally injected with 100 ng of PMA, the PKC-βII agonist; (v) the 8-pCPT group: rats were intraperitoneally injected with 500 ng of8-pCPT, agonist for Epac1; (vi) the SMIR group: rats received SMIR surgery; (vii) the SMIR + Ad-sh-NC group: rats received SMIR surgery after injection of adenovirus expressing negative control short hairpin RNA; (viii) the SMIR + Ad-sh-Epac1 group: rats received SMIR surgery after injection of adenovirus expressing short hairpin RNA against Epac1; (ix) the SMIR + LY333531 group: rats received SMIR surgery and injection of 1 mg/kg LY333531 (Eli Lilly, Indianapolis, IN). LY333531 is selective inhibitors of conventional protein kinase C; (x) the SMIR + saline group: rats were intraperitoneally injected with 20 mg/kg/day normal saline for 14 days before the SMIR surgery; (xi) the SMIR + Captopril group: rats were intraperitoneally injected with 20 mg/kg/day Captopril for 14 days before the SMIR surgery. Captopril is an angiotensin-converting enzyme (ACE) inhibitor with antioxidant and anti-inflammatory properties which has been suggested as the preferred agents for treatment on pain modulation [20]. Here we used Captopril to protect vascular endothelial cells after SMIR and have a positive effect on postoperative pain

### Von Frey filament testing

Hind paw allodynia was assessed by von Frey filaments (Bioseb, Chaville, France). Rats were accommodated in a small plastic cage (35 × 20 × 15 cm) for 1 h. Starting with the lowest force, each filament was applied 3 times with intervals of 3 seconds. The stimulation was terminated upon either a brisk paw withdrawal and/or an escape attempt, and the force was recorded as the mechanical response threshold. The cut-off threshold was set as 30 g.

### Isolation and culture of the DRG (DRG) neurons

Dorsal root ganglia (L3-L5) were harvested 1 hr, removed from rats, and dissected in an oxygenated ice-cold dissecting solution. After incubation in the dissecting solution supplemented with 1 mg/mL trypsin and collagenase D at room temperature for 1 h, dorsal root ganglia were removed from the solution, washed, and dissociated by trituration. Cells were cultured in Neuro Basal-A medium with 2% B27 supplement, 2 mM GlutaMAX, and antibiotics at the ambient temperature of 5% CO_2_ and 80% humidity. The next day after incubation, cells were treated with 20 mM 5-fluorodeoxyuridine and uridine for 3 days to remove non-neural cells.

### Cell treatment

DRG neurons were assigned into six groups: (i) the control group; (ii) cells were treated with 0.25% DMSO; (iii) cells were treated with 100 μM 8-pCPT; (iv) cells were treated with 50 ng/mL PMA; (v) cells were transfected with sh-Epac1 (GenePharma) using the lipofectamine 3000 (Invitrogen) for 48 h and then treated with 100 μM 8-pCPT; (vi) cells were cotreated with 50 ng/mL PMA and 0.5 μM LY333531.

### Reverse transcription-quantitative PCR

Total RNA was extracted from DRG cells using TRIzol reagent (Invitrogen) according to the manufacturer’s instructions and the RNA purity was determined by the SmartSpec Plus Spectrophotometer (Bio-Rad Laboratories). A Transcriptor First Strand cDNA synthesis kit (Roche) was used for the synthesis of the cDNA. PCR was conducted on an ABI 7500 real-time PCR system using SYBR Green PCR Master Mix (both from Applied Biosystems) following the manufacturer’s protocol. NF200 and NSE mRNA expression levels were calculated using the 2(-Delta Delta C(T)) method [21] and were normalized to internal control Actin. Primer sequences used in PCR analysis are listed below:

NF200: forward, 5’-GTTCCGAGTGAGATTGGAC-3’;

reverse, 5’-GTTATCTCCTCTTGGGCAG-3’.

NSE: forward, 5’-CGGAACTATCCTGTGGTCTC-3’;

reverse, 5’-GACATTGGCTGTGAACTTGG-3’.

TNF-α: forward, 5’-CTTCTCATTCCTGCTCGTG-3’;

reverse, 5’-TTTGGGAACTTCTCCTCCT-3’.

IL-1β: forward, 5’-TTCATCTTTGAAGAAGAGCCC-3’;

reverse, 5’-CTGTCTAATGGGAACATCACAC-3’.

MCP-1: forward, 5’-GAAGCTGTAGTATTTGTCACCA-3’;

reverse, 5’-TCTAATGTACTTCTGGACCCA-3’.

Actin: forward, 5’-CTTCCTGGGTATGGAATCCT-3’;

reverse, 5’-TCTTTACGGATGTCAACGTC-3’.

### Western blotting

After culturing for 48 h, Western blotting was performed on DRG neurons. Proteins (60 μg) were separated by sodium dodecyl sulfate-polyacrylamide gel electrophoresis (SDS-PAGE) and then transferred onto a polyvinylidene difluoride membrane. The membrane was blocked with 5% skim milk powder and incubated with primary antibodies to Epac1 (1:1000, ab124162), p-PKCβII (1:1000, #9375), PKCβII (1:3000, ab32026), Glut1 (1:100000, ab115730), VEGF (1:1000, ab1316), EPO (1:5000, ab226956) or PGP9.5 (1:1000, ab108986), PCNA (1:1000, ab92552), Cyclin D1 (1:200, ab16663), and the loading control β-actin (1:5000, ab8227) at 4°C overnight. On the next day, the membrane was incubated with horseradish peroxidase-conjugated goat anti-rabbit IgG (1:2000, ab6721). Antibodies were purchased from Abcam (Shanghai, China) or Cell Signaling TECHNOLOGY. Finally, membranes were exposed to an Enhanced Chemiluminescence Detection Kit (Thermo, Waltham, MA) for protein visualization. The gray density of the protein bands was quantified with the ImageJ software.

### Cell viability assay

Cell viability was assessed by the 3-(4,5-dimethyl-2-thiazolyl)-2,5-diphenyl-2H-tetrazolium bromide (MTT, Sigma) assay. DRG neurons were seeded into a 96-well plate at the density of 1×10^4^ cells/well for 12, 24, and 48 h. Next, the media was replaced with the media containing 0.5 mg/mL MTT and cells were incubated at ambient temperature for another 4 h. Formazan crystals were solubilized by dimethyl sulfoxide, and cell absorbance was measured at 570 nm.

### Statistical analysis

Data from three technical repeats are presented as the mean ± SEM. Differences between 2 mean values were analyzed with the Student unpaired *t*-test. Comparisons between multiple mean values were performed with one-way ANOVA and the Tukey’s *post hoc* test or two-way ANOVA. *P* values less than 0.05 were statistically significant.

## Results

### Effects of SMIR surgery on the Epac/PKCβII pathway, mechanical hypersensitivity in rats

**Fig. 1:**
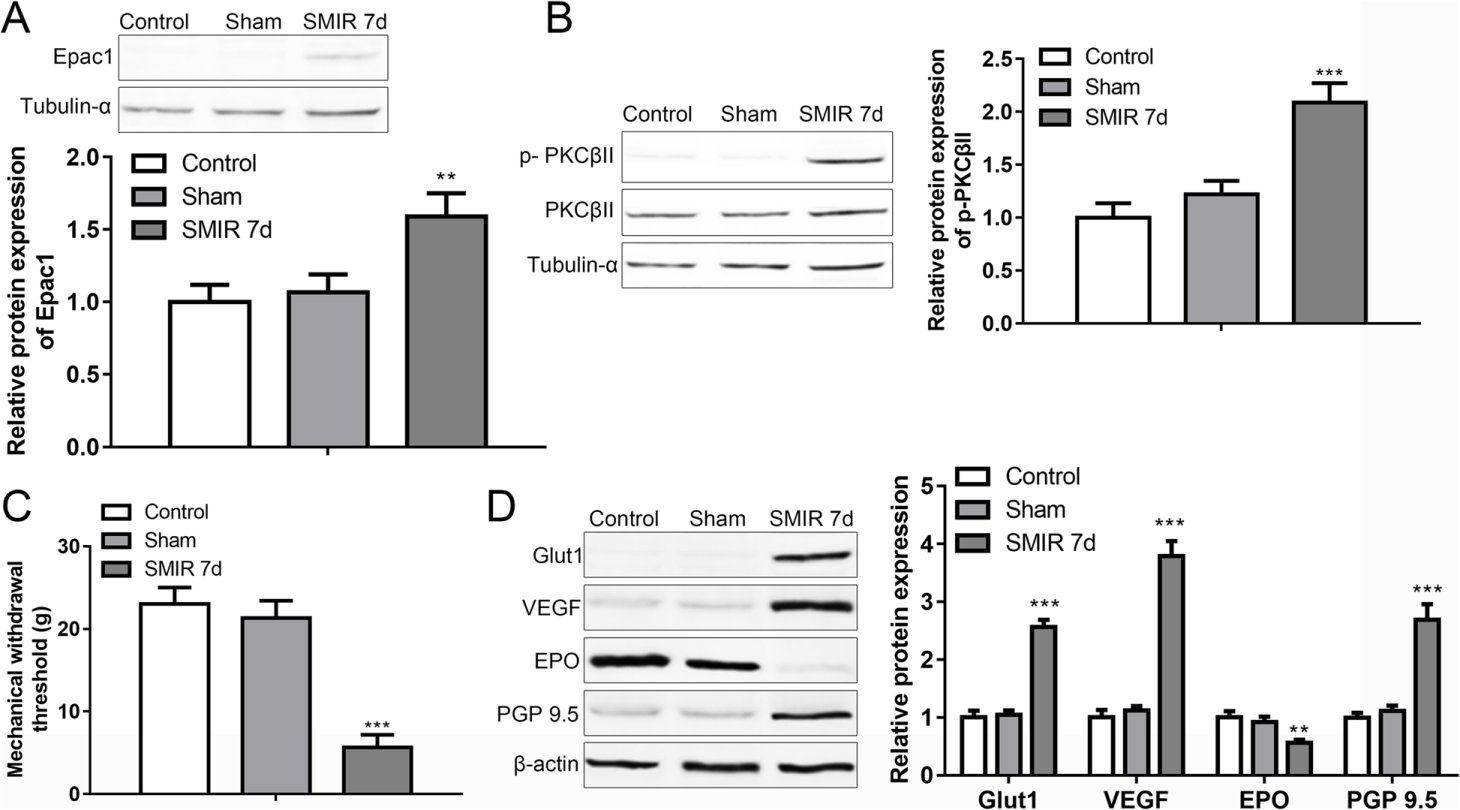
Effects of SMIR surgery on the Epac/PKCβII pathway, mechanical hypersensitivity in rats. **A** Epac1 protein level in DRG of rats in the control, sham, SMIR 7d groups was revealed by western blotting analysis. **B** Western blotting analysis of p-PKCβII and PKCβII proteins in DRG of rats in the three groups. **C** Mechanical withdrawal threshold of rats in the three groups. **D** Glut1, VEGF, PGP9.5, and EPO proteins in DRG of rats were assessed by Western blotting. **p < 0.01, ***p < 0.001 vs. control. (Full-length blots are presented in Supplementary File.)

Relative protein expression of Epac1 was higher in the DRGs from rats after SMIR for seven days than DRGs from control rats (Fig. 1A). Expression of p-PKCβII was also higher in the DRGs from rats after SMIR for seven days than control DRGs (Fig. 1B). SMIR surgery significantly evoked the mechanical hypersensitivity in the hind paw of rats (Fig. 1C). Moreover, Glut1, VEGF, and PGP9.5 proteins in DRGs were increased, while EPO protein was decreased by SMIR after 7 days (Fig. 1D).

### The Epac/PKCβII pathway mediates inflammatory microenvironment and mechanical hypersensitivity in rats after SMIR

**Fig. 2:**
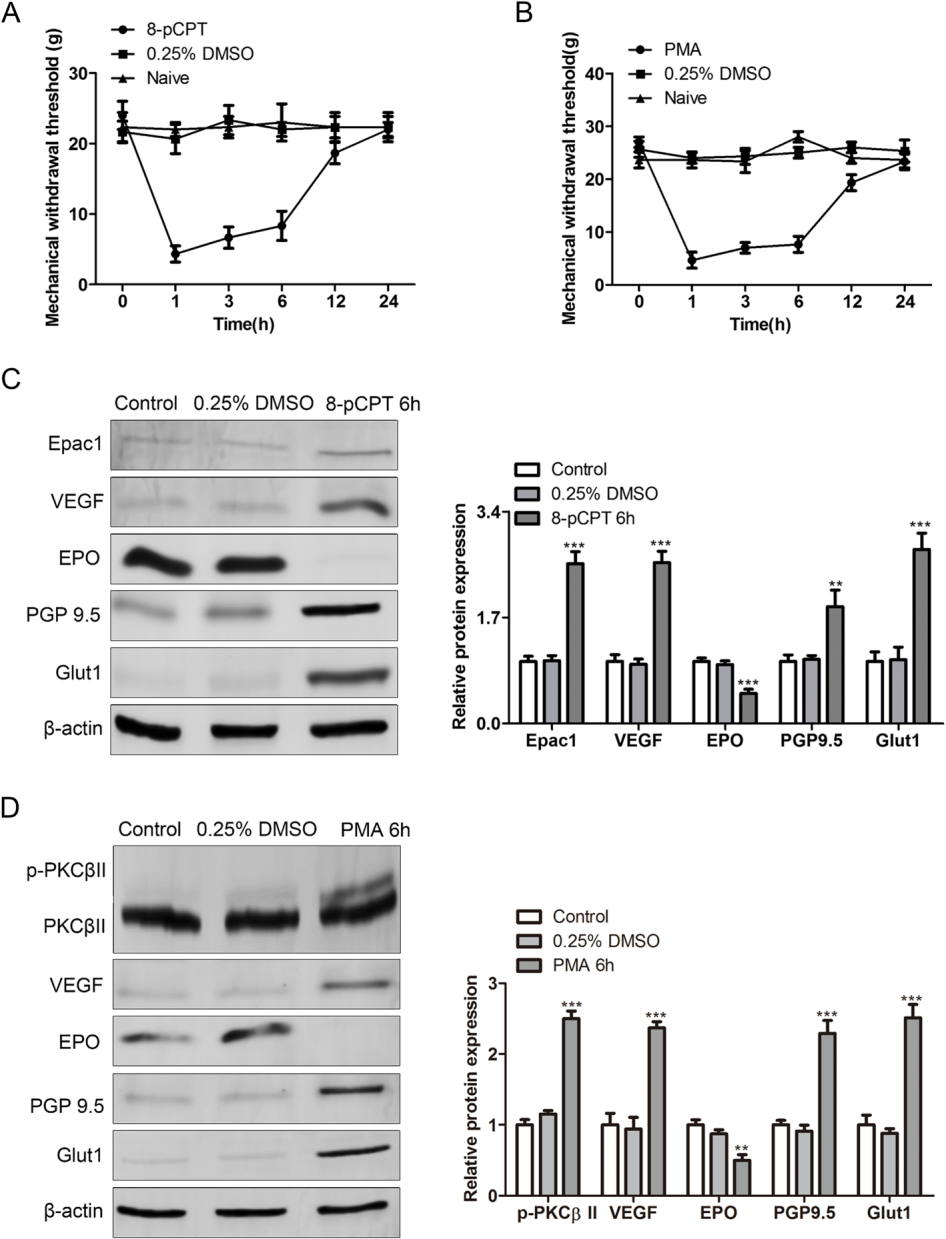
The Epac/PKCβII pathway mediates inflammatory microenvironment and mechanical hypersensitivity in rats after SMIR. **A** Mechanical withdrawal threshold of rats of the control, 0.25% DMSO, and 8-pCPT groups. **B** Mechanical withdrawal threshold of rats of the control, 0.25% DMSO, and PMA groups. **C** Epac1, VEGF, EPO, PGP9.5, and Glut1 proteins in DRG of rats of the control, 0.25% DMSO, and 8-pCPT groups. **D** p-PKCβII, PKCβII, VEGF, EPO, PGP9.5, and Glut1 proteins in DRG of rats of the control, 0.25% DMSO, and PMA groups. **p < 0.01, ***p < 0.001 vs. control

After the injection of 8-pCPT (agonist for Epac1) (Fig. 2A) or PMA (agonist for PKCβII) (Fig. 2B), SMIR-operated rats exhibited the decreased mechanical withdrawal threshold. 8-pCPT treatment caused the upregulation of Epac1, Glut1, VEGF, and PGP9.5 proteins as well as the downregulation of EPO protein in the SMIR-operated rats (Fig. 2C). Furthermore, PMA treatment caused the increase of p-PKCβII, Glut1, VEGF, PGP9.5 proteins and the decrease of EPO protein in the SMIR-operated rats (Fig. 2D).

### Suppression of the Epac/PKCβII pathway rescues the SMIR-induced inflammatory microenvironment and mechanical hypersensitivity

**Fig. 3:**
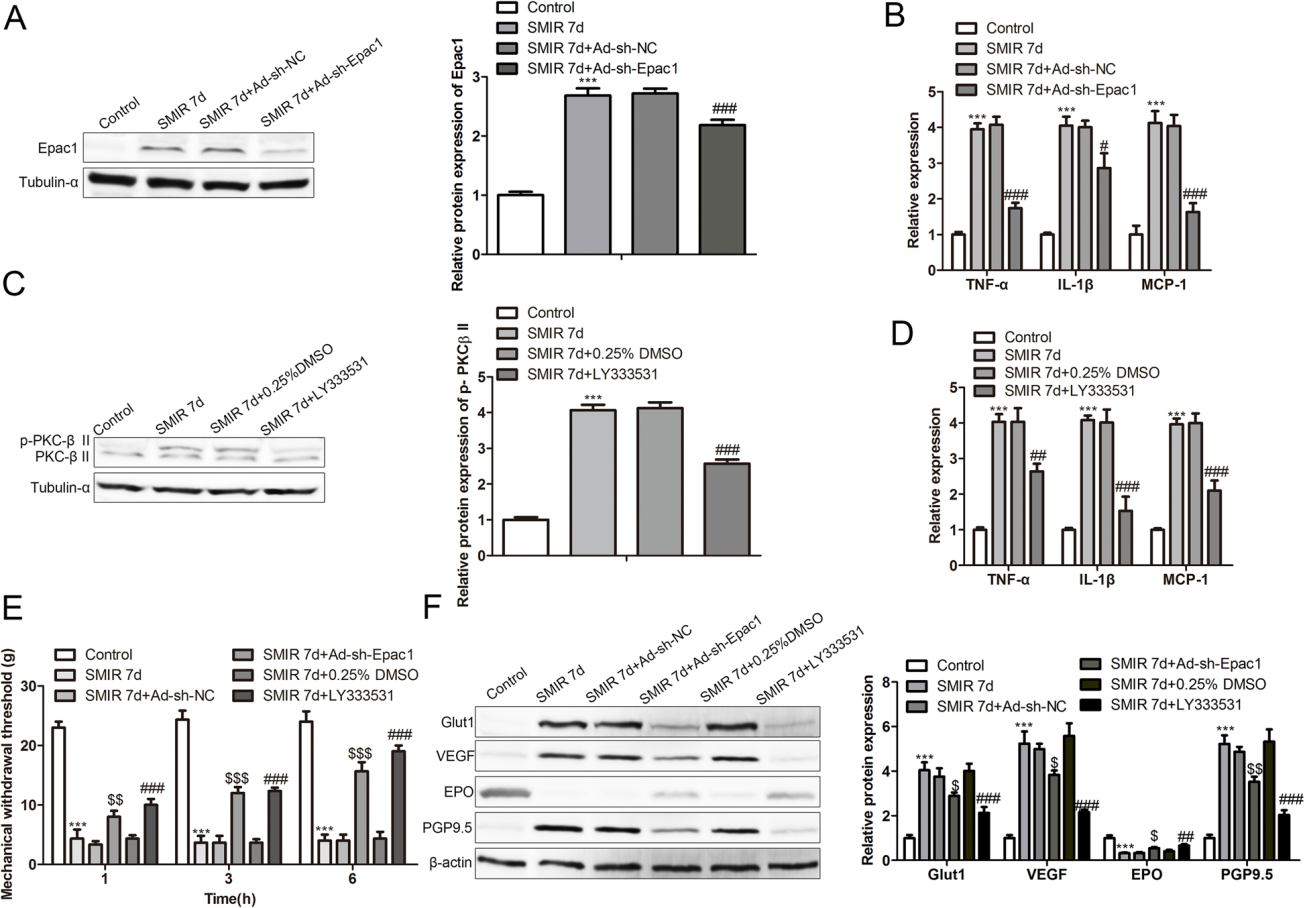
Suppression of the Epac/PKCβII pathway rescues the SMIR-induced inflammatory microenvironment and mechanical hypersensitivity. **A** Epac1 protein level in DRG of rats in the control, SMIR, SMIR + Ad-sh-NC, and SMIR + Ad-sh-Epac1 groups was revealed by western blotting analysis. **B** TNF-α, IL-1β, and MCP-1 mRNA levels in DRG of rats. ***p < 0.001 vs. control, ^#^ *p* < 0.05, ^###^ *p* < 0.001 vs. SMIR + Ad-sh-NC. **C** p-PKCβII and -PKCβII protein levels in DRG of rats in the control, SMIR, SMIR + 0.25% DMSO, and SMIR + LY333531 groups were revealed by western blotting analysis. **D** TNF-α, IL-1β, and MCP-1 mRNA levels in DRG of rats. ***p < 0.001 vs. control, ^##^*p* < 0.01 , ^###^ *p* < 0.001 vs. SMIR + 0.25% DMSO. **E** Mechanical withdrawal threshold of rats in the control, SMIR, SMIR + Ad-sh-NC, SMIR + Ad-sh-Epac1, SMIR + 0.25% DMSO, and SMIR + LY333531 groups. **F** Glut1, VEGF, PGP9.5, and EPO proteins in DRG of rats. ***p < 0.001 vs. control, ^$^ *p* < 0.05, ^$$^ *p* < 0.01, ^$$$^ *p* < 0.001 vs. SMIR + Ad-sh-NC, ^##^*p* < 0.01 , ^###^ *p* < 0.001 vs. SMIR + 0.25% DMSO

After injection of Ad-sh-Epac1, SMIR-operated rats showed decreased Epac1 expression in the DRG (Fig. 3A). Compared with the rats with only SMIR operation, SMIR-operated rats with adenovirus-mediated Epac1 deficiency showed the lower mRNA expression of proinflammatory factors including TNF-α, IL-1β, and MCP-1 in the DRG (Fig. 3B). Administration of LY333531 caused the inhibition of p-PKCβII protein (Fig. 3C) and induced the decrease of TNF-α, IL-1β, and MCP-1 levels (Fig. 3D) in the DRG of SMIR-operated rats. Administration of Ad-sh-Epac1 or LY333531 decreased mechanical withdrawal threshold in rats after SMIR operation for seven days (Fig. 3E). Figure 3F revealed that suppression of Epac1 and PKCβII decreased the Glut1, VEGF, PGP9.5 proteins and increased the EPO protein in the DRG of rats after SMIR operation for seven days.

### The Epac/PKCβII pathway promotes the abnormal proliferation of the DRG cells

**Fig. 4:**
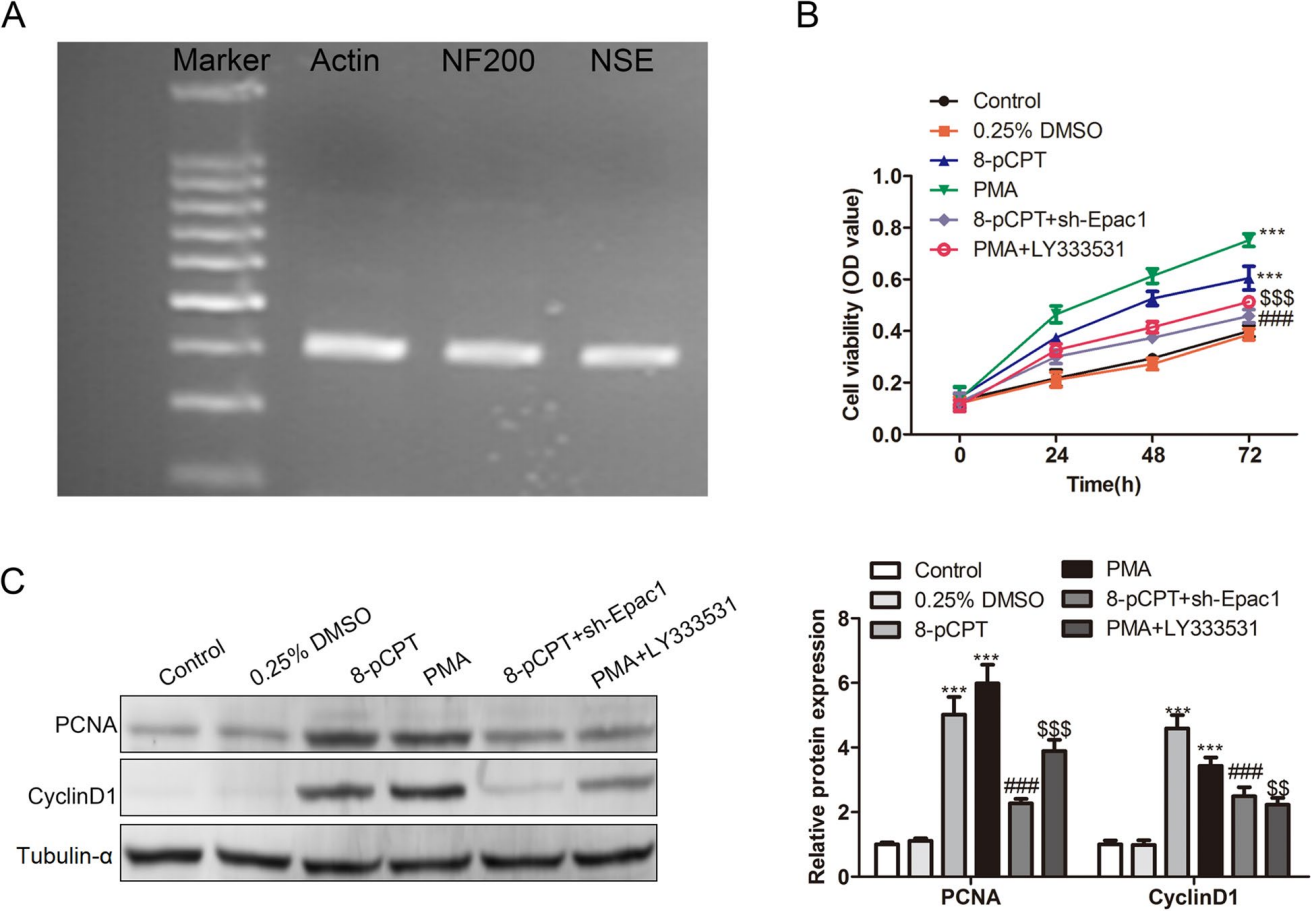
The Epac/PKCβII pathway promotes the abnormal proliferation of the DRG cells. **A** PCR analysis of NF200 and NSE to confirm the isolated DRG cells. **B** Viability of DRG cells of the six groups: control, 0.25% DMSO, 8-pCPT, PMA, 8-pCPT+sh-Epac1, PMA+LY333531 was detected by MTT assay. **C** Protein levels of PCNA and CyclinD1 in DRG cells of the six groups. ***p < 0.001 vs. control, ^###^ *p* < 0.001 vs. 8-pCPT, ^$$$^ *p* < 0.001 vs. PMA

Primary DRG cells were isolated and confirmed by RT-qPCR, as evidenced by the presence of NF200 and NSE (Fig. 4A). After treatment with 8-pCPT and PMA, the cell viability of DRG cells was increased, while sh-Epac1 rescued the effect of 8-pCPT, and LY333531 rescued the effect of PMA on cell viability (Fig. 4B). 8-pCPT or PMA treatment also enhanced the protein levels of PCNA and CyclinD1, and sh-Epac1 and LY333531 rescued the effect of 8-pCPT and PMA on these proteins, respectively (Fig. 4C).

### Protection of vascular endothelial cells on mechanical hypersensitivity and MCP-1 expression

**Fig. 5:**
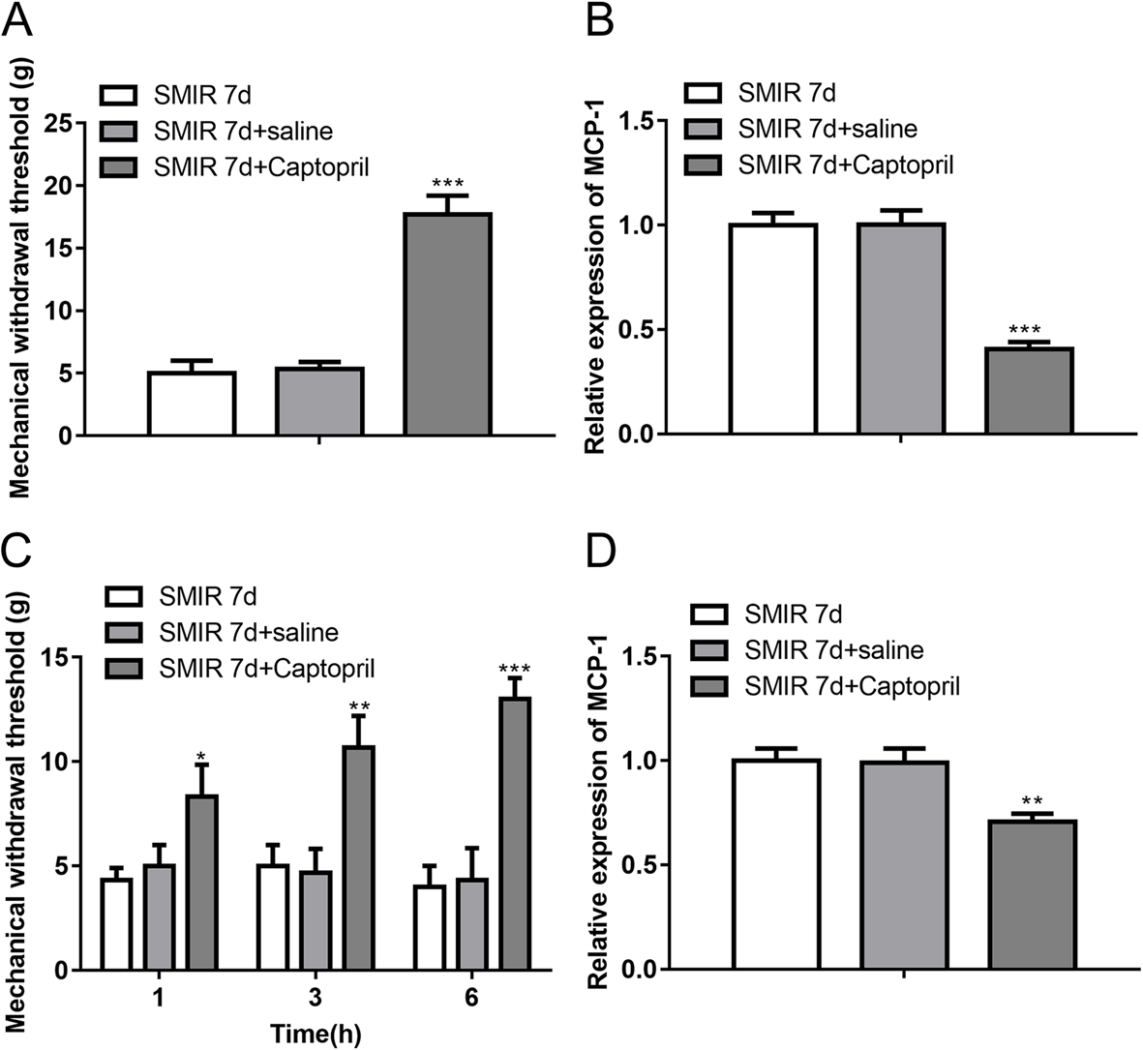
Protection of vascular endothelial cells on mechanical hypersensitivity and MCP-1 expression. **A** Mechanical withdrawal threshold of rats with SMIR operation for 7 days, with SMIR operation for 7 days plus pretreatment of saline, or with SMIR operation for 7 days plus pretreatment of Captopril. **B** Relative mRNA expression of MCP-1 in the DRG of rats. **C** Mechanical withdrawal threshold of rats with SMIR operation for 7 days, with SMIR operation for 7 days followed by saline administration, or with SMIR operation for 7 days followed by Captopril administration. **D** Relative mRNA expression of MCP-1 in the DRG of rats. **p < 0.01, ***p < 0.001 vs. SMIR 7d+saline

Pretreatment with Captopril enhanced mechanical withdrawal threshold (Fig. 5A) and decreased MCP-1 expression in DRG (Fig. 5B) in rats after SMIR operation for seven days. Figure 5C, D indicated that treatment of Captopril after SMIR operation for seven days also increased mechanical withdrawal threshold (Fig. 5C) and reduced MCP-1 expression in DRG (Fig. 5D) in rats.

## Discussion

Management of postoperative pain is hindered by the poor understanding of the pathogenesis underlying postoperative pain [22]. Surgery-induced increase of proinflammatory factors in DRG exerts an essential role in generating neuronal hypersensitivity and thus contributing to postoperative pain [23]. In the present study, we found that SMIR induced the upregulation of TNF-α, IL-1β, and MCP-1 in the DRG in rats, which is consistent with the previous studies. Spinal Epac1 expression was found to increase in an animal model of postoperative pain which might be caused by central sensitization in the spinal cord [24]. In our study, we proved the molecular changes in the DRG, indicating peripheral sensitization, which is not totally the same as central sensitization. The NMDAR/AC1/cAMP/Epac pathway in the dorsal horn maintains latent pain sensitization in both sexes [25]. Increased PKC-dependent phosphorylation of GluN1 contributes to mechanical allodynia in a chronic constriction injury-induced mouse model of neuropathic pain [26]. Increased PKC was found in postoperative DRG, and PKC, but not PKA, promotes postoperative hyperalgesia [27]. In the present study, after SMIR for seven days, the Epac1 and PKCβII proteins were increased in DRG in rats, confirming the pivital role of the Epac1/PKC pathway in postoperative pain.

Glut1 expression is positively correlated with microvessel density in postoperative pain [28, 29]. SMIR surgery induces the increased Glut1 protein around the incision site in rats [30]. PKCβII promotes PGP9.5 and VEGF proteins in DRG of the SMIR-operated rats [31]. PGP9.5 is a nerve-specific marker, and an increase or decrease of its expression is associated with nerve repair or damage, respectively [32]. The PGP9.5 expression is increased during the inflammation and pain process [33, 34]. VEGF can increase vascular permeability [35] and glucose levels in the cell by PKC [36], and protect nerve cells [37]. Increased VEGF induces an inflammatory response and facilitates the release of algogenic substances [38]. EPO is a hematopoietic hormone, exerting anti-inflammatory and neuroprotective effects [39], and has been indicated to prevent postoperative cognitive dysfunction [40]. The present study revealed that SMIR promoted Glut1, VEGF, PGP9.5 protein levels, and decreased EPO protein expression in DRG of rats with postoperative pain, which was in accordance with the previous studies. Pretreatment with Captopril in SMIR rats reduced mechanical hypersensitivity and MCP-1 expression, indicating the positive effect of vascular protection in postoperative pain.

In some animal studies, the Epac1 agonist and PKCβII agonist were used to treat the normal rats. These agonists induced hyperalgesia and increased Glut1, VEGF, PGP9.5 protein levels, as well as decreased EPO protein level in DRG. After the suppression of the Epac/PKCβ pathway, SMIR rats showed alleviated hyperalgesia, decreased TNF-α, IL-1β, MCP-1, Glut1, VEGF, PGP9.5 levels and increased EPO level. These findings revealed that the Epac/PKCβ pathway plays a significant role in postoperative pain, and its effects were associated with inflammation and BNB function.

However, the detailed mechanism by which the Epac/PKCβ pathway regulates postoperative pain was not explored in the present study. A previous study has indicated the calcium influx after activation of the Epac/PKC pathway [41], which encourages us to study calcium channel in the future. Besides, a study showed that NMDAR→AC1→cAMP→Epac signaling pathways in the dorsal horn of the spinal cord maintain latent sensitization in both males and females, which presents a novel mechanism for the promotion of chronic postoperative pain [25]. Whether the Epac/PKCβ pathway is activated in other tissues during the SMIR-induced postoperative pain remains unknown and requires further investigation. Moreover, inflammatory communication between neurons and gliocyte as well as the polarization of glial cells are essential in SMIR-induced post-surgical pain [42]. Our further research will focus on neuron-glial communication and glial activation in post-surgical pain.

To sum up, our findings provided evidence that SMIR induces the Epac/PKCβ pathway in the DRG. This pathway influences inflammation and BNB function and regulates mechanical hypersensitivity in rats with postsurgical pain, indicating that targeting the Epac/PKCβ pathway can help treat postoperative pain.

## Supplementary Information

**Additional file 1.**

## Data Availability

The raw data supporting the conclusions of this manuscript will be made available by the authors, without undue reservation, to any qualified researcher.
