# Supplementary material for: Skin/muscle incision and retraction regulates the persistent postoperative pain in rats by the Epac1/PKC-βII pathway
Source: BMC Anesthesiol. 2022 Jul 18;22:230. doi: 10.1186/s12871-022-01771-w (PMC9290233; doi:10.1186/s12871-022-01771-w)
Supplement: Supplementary file 1 — Additional file 1. [file 12871_2022_1771_MOESM1_ESM.pptx]

## Slide 1
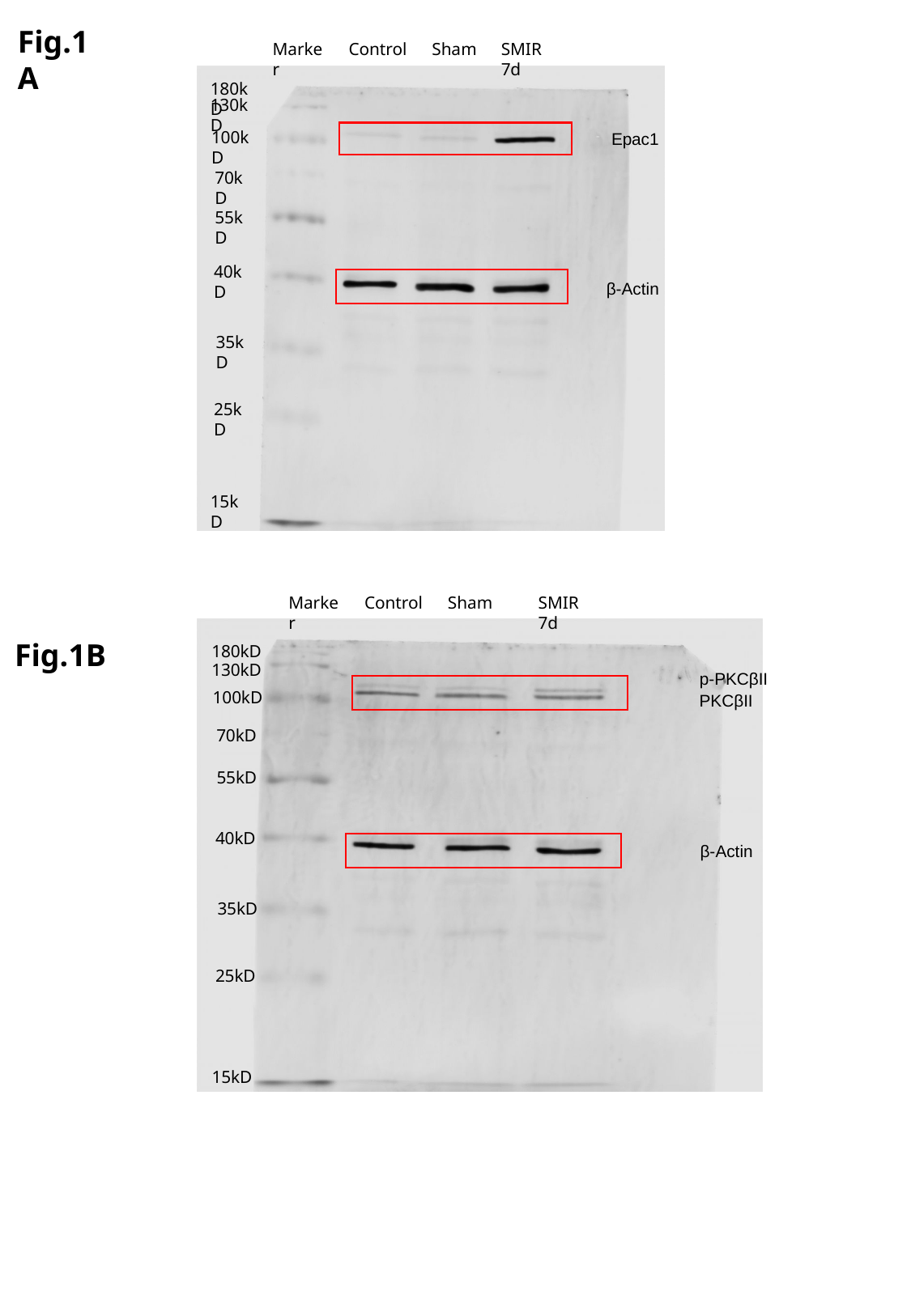

Fig.1A
Marker
Sham
Control
SMIR 7d
180kD
130kD
100kD
Epac1
70kD
55kD
40kD
β-Actin
35kD
25kD
15kD
Marker
Sham
Control
SMIR 7d
Fig.1B
180kD
130kD
p-PKCβII
100kD
PKCβII
70kD
55kD
40kD
β-Actin
35kD
25kD
15kD

## Slide 2
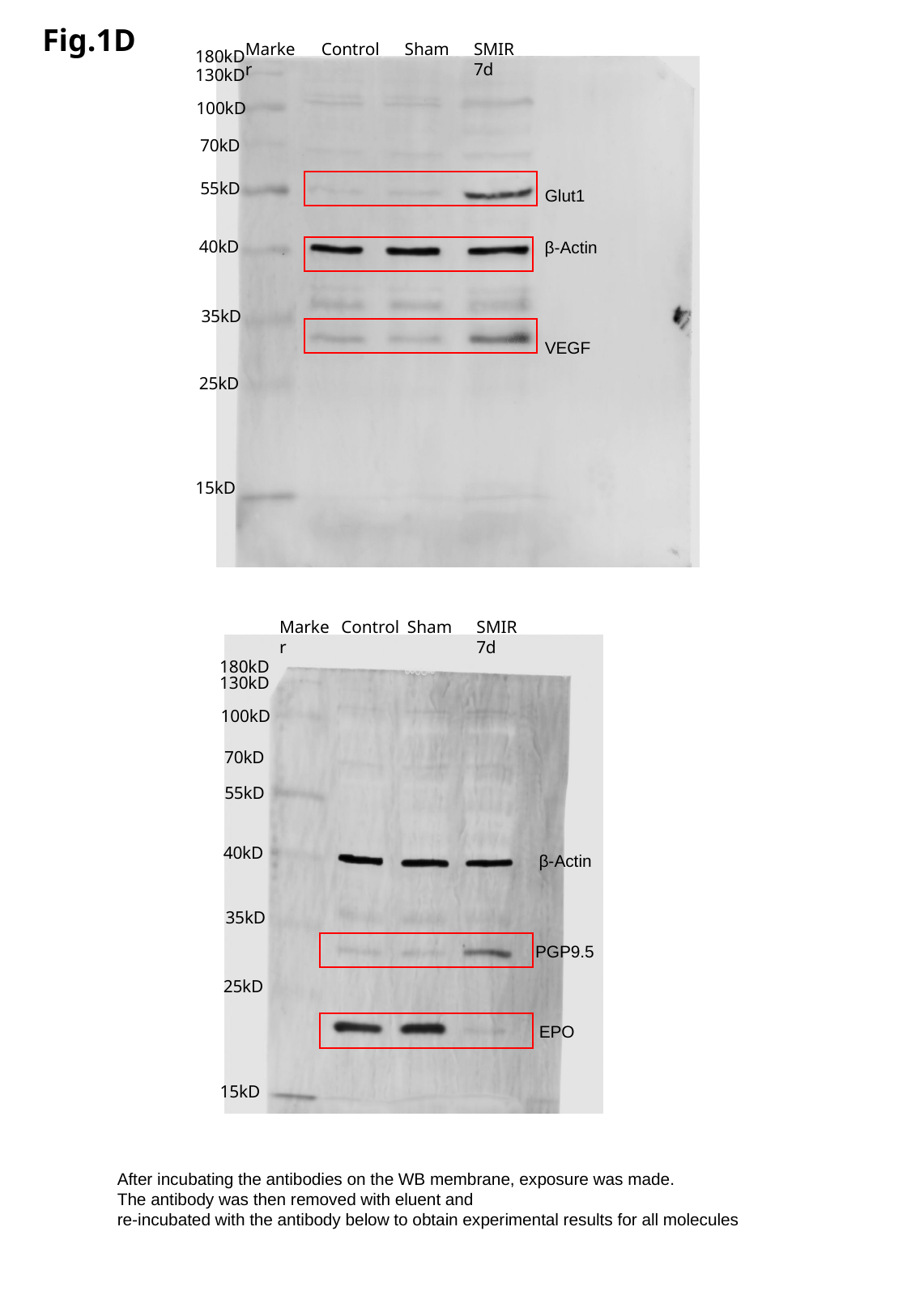

Fig.1D
Marker
Sham
Control
SMIR 7d
180kD
130kD
100kD
70kD
55kD
Glut1
40kD
β-Actin
35kD
VEGF
25kD
15kD
Marker
Sham
Control
SMIR 7d
180kD
130kD
100kD
70kD
55kD
40kD
β-Actin
35kD
PGP9.5
25kD
EPO
15kD
After incubating the antibodies on the WB membrane, exposure was made.
The antibody was then removed with eluent and
re-incubated with the antibody below to obtain experimental results for all molecules

## Slide 3
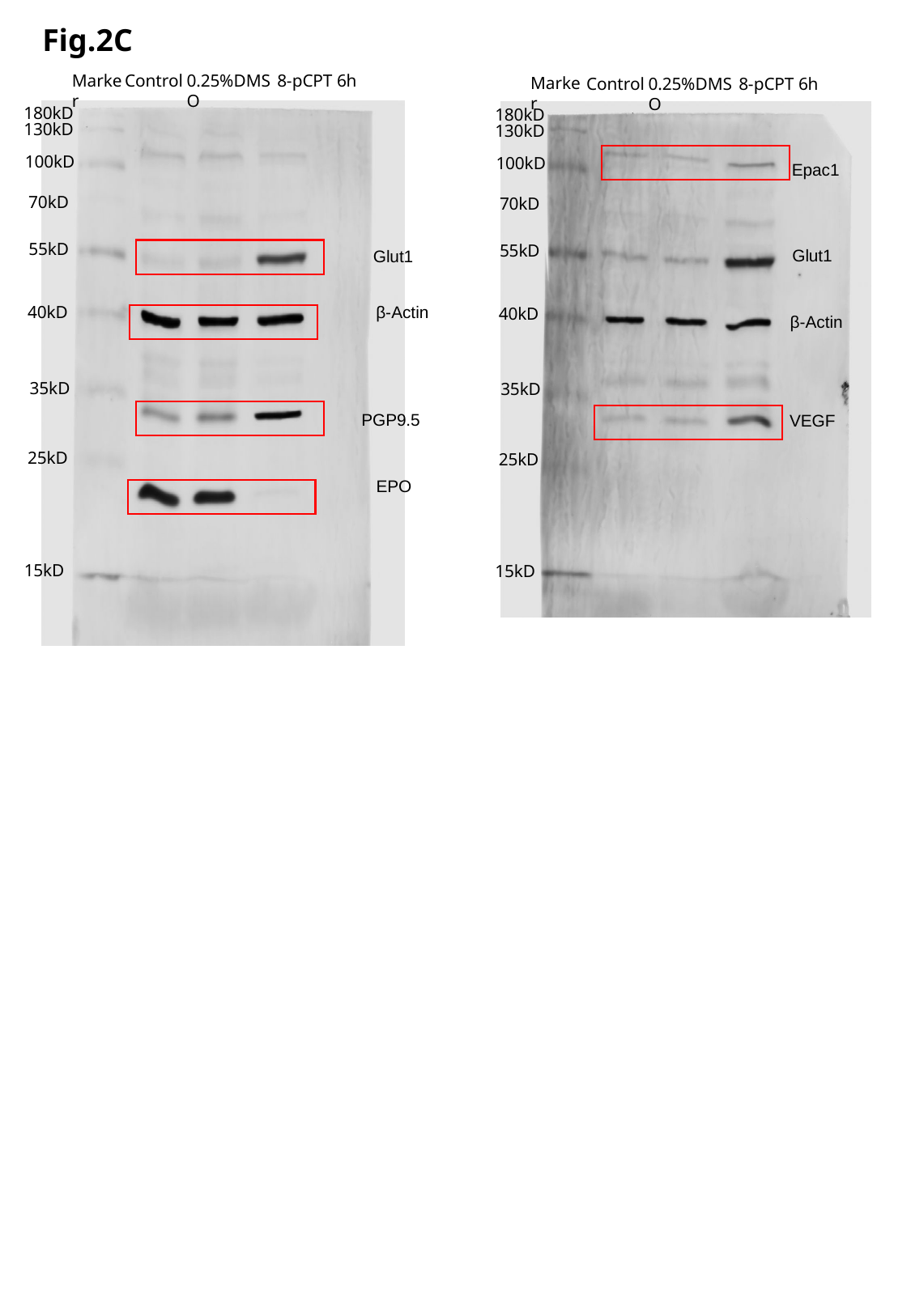

Fig.2C
Marker
Control
0.25%DMSO
8-pCPT 6h
Marker
Control
0.25%DMSO
8-pCPT 6h
180kD
180kD
130kD
130kD
100kD
100kD
Epac1
70kD
70kD
55kD
55kD
Glut1
Glut1
40kD
β-Actin
40kD
β-Actin
35kD
35kD
PGP9.5
VEGF
25kD
25kD
EPO
15kD
15kD

## Slide 4
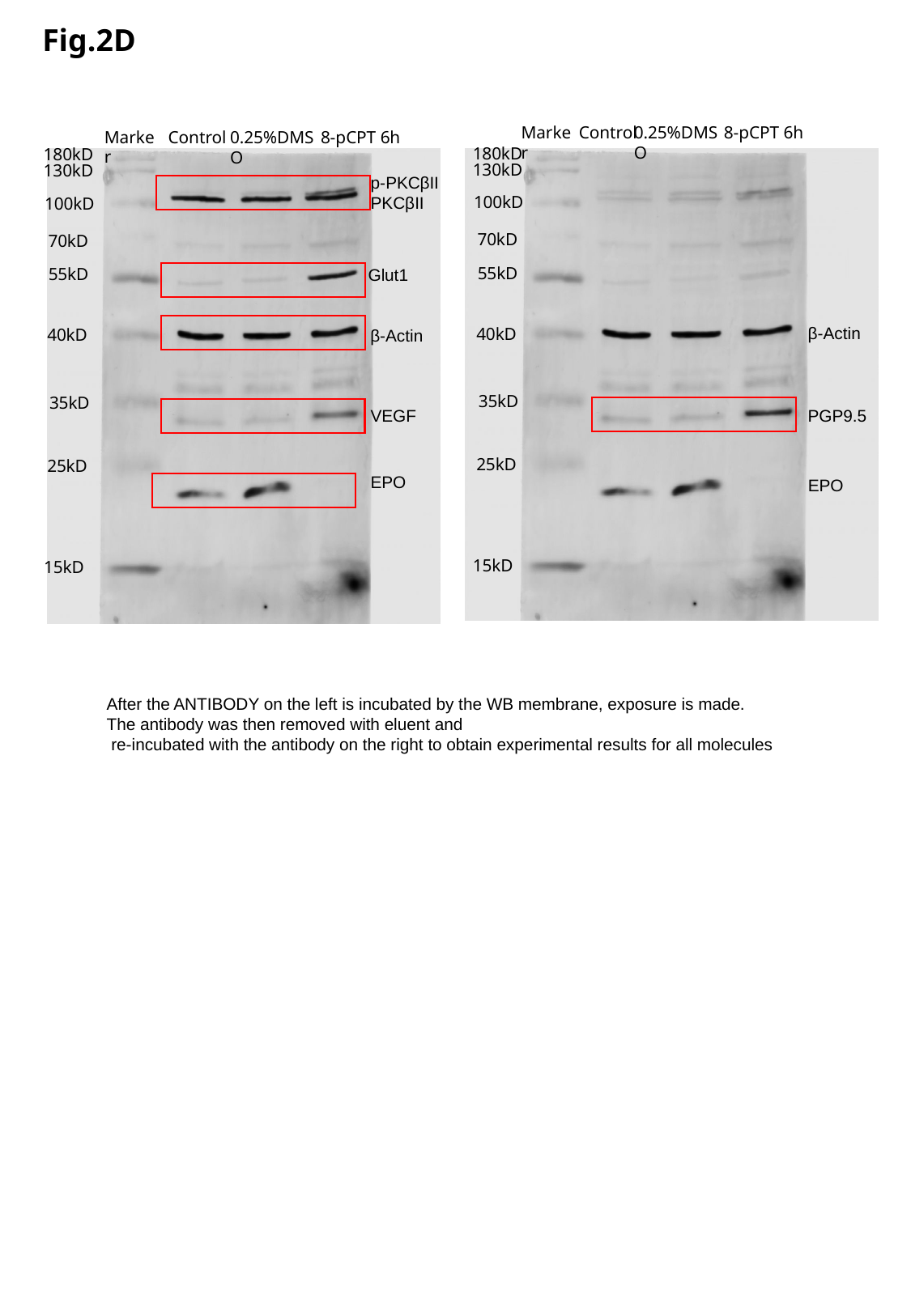

Fig.2D
Marker
Control
0.25%DMSO
8-pCPT 6h
Marker
Control
0.25%DMSO
8-pCPT 6h
180kD
180kD
130kD
130kD
p-PKCβII
PKCβII
100kD
100kD
70kD
70kD
55kD
55kD
Glut1
β-Actin
40kD
40kD
β-Actin
35kD
35kD
VEGF
PGP9.5
25kD
25kD
EPO
EPO
15kD
15kD
After the ANTIBODY on the left is incubated by the WB membrane, exposure is made.
The antibody was then removed with eluent and
 re-incubated with the antibody on the right to obtain experimental results for all molecules

## Slide 5
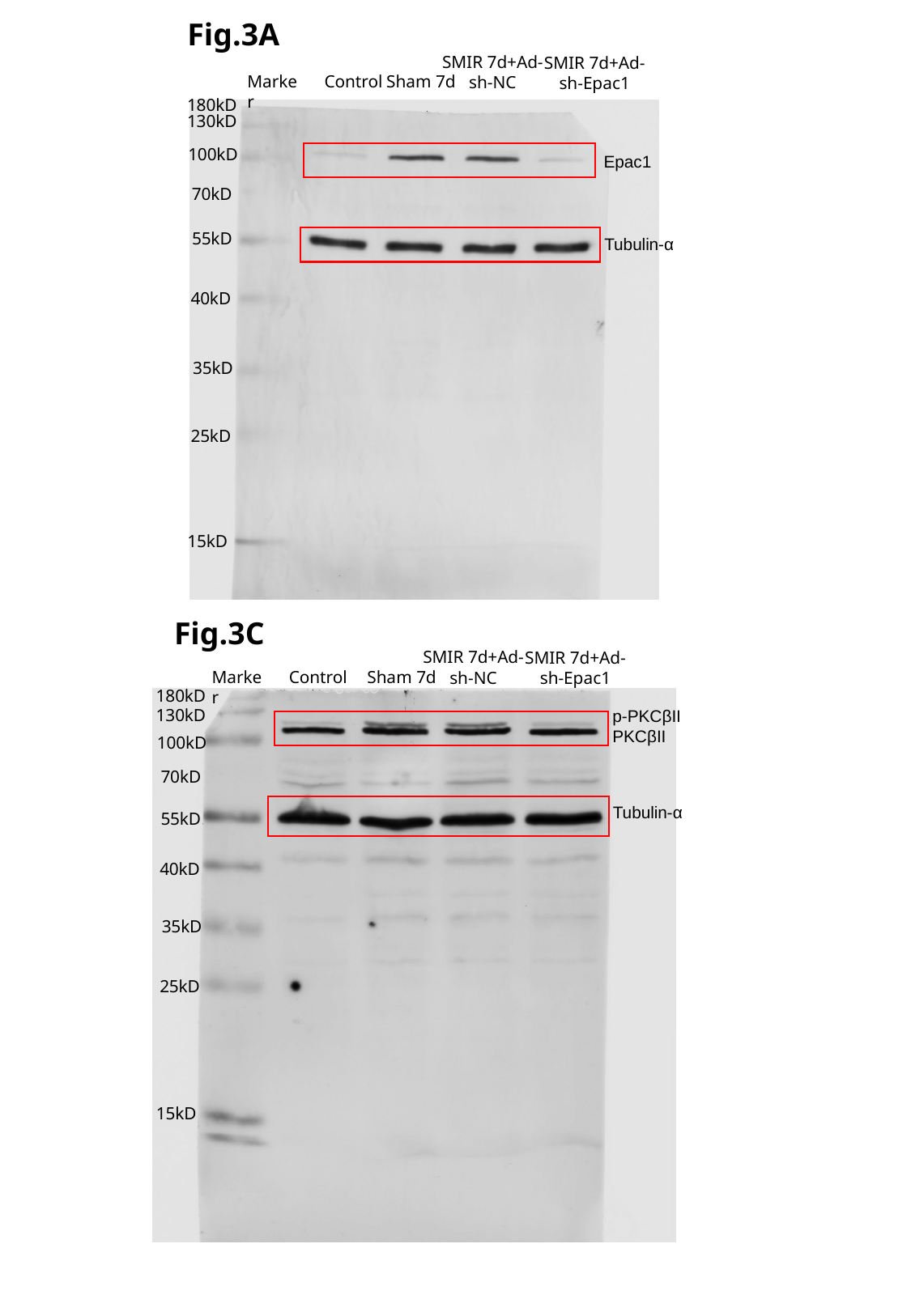

Fig.3A
SMIR 7d+Ad-sh-NC
SMIR 7d+Ad-sh-Epac1
Marker
Control
Sham 7d
180kD
130kD
100kD
Epac1
70kD
55kD
Tubulin-α
40kD
35kD
25kD
15kD
Fig.3C
SMIR 7d+Ad-sh-NC
SMIR 7d+Ad-sh-Epac1
Marker
Control
Sham 7d
180kD
130kD
p-PKCβII
PKCβII
100kD
70kD
Tubulin-α
55kD
40kD
35kD
25kD
15kD

## Slide 6
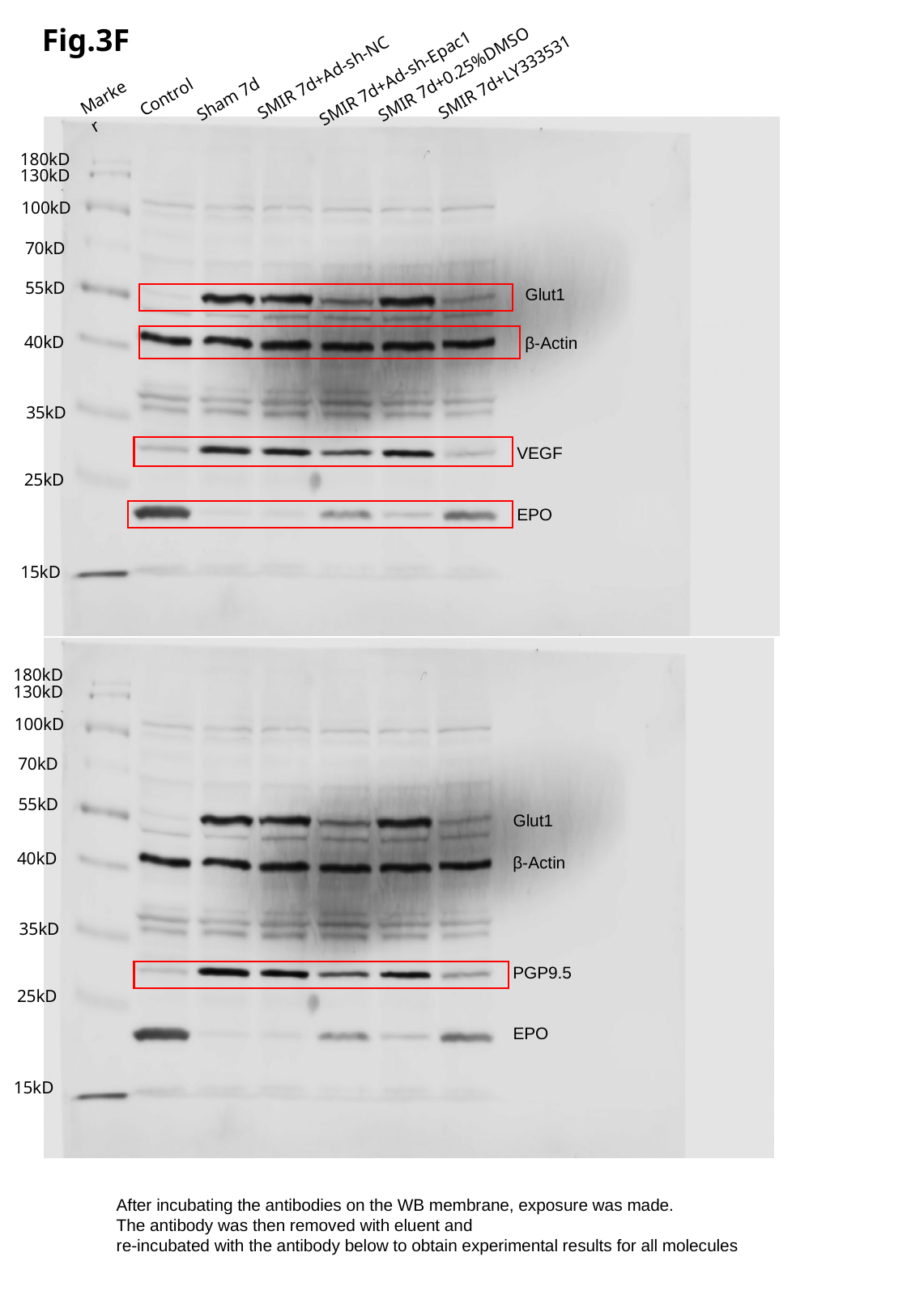

Fig.3F
SMIR 7d+0.25%DMSO
SMIR 7d+LY333531
SMIR 7d+Ad-sh-NC
SMIR 7d+Ad-sh-Epac1
Marker
Control
Sham 7d
180kD
130kD
100kD
70kD
55kD
Glut1
40kD
β-Actin
35kD
VEGF
25kD
EPO
15kD
180kD
130kD
100kD
70kD
55kD
Glut1
40kD
β-Actin
35kD
PGP9.5
25kD
EPO
15kD
After incubating the antibodies on the WB membrane, exposure was made.
The antibody was then removed with eluent and
re-incubated with the antibody below to obtain experimental results for all molecules

## Slide 7
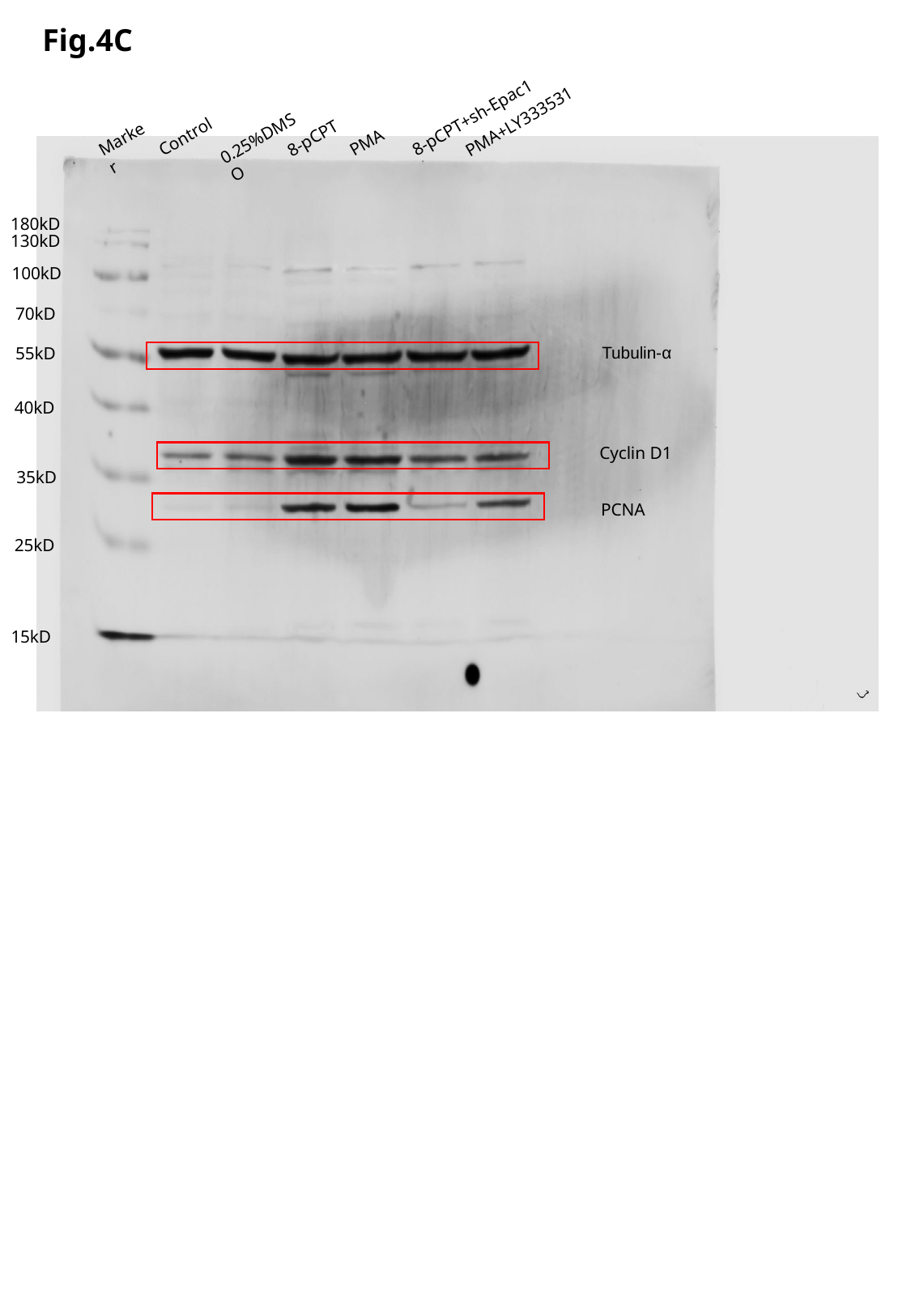

Fig.4C
8-pCPT+sh-Epac1
PMA+LY333531
PMA
0.25%DMSO
8-pCPT
Control
Marker
180kD
130kD
100kD
70kD
Tubulin-α
55kD
40kD
Cyclin D1
35kD
PCNA
25kD
15kD
